# Evaluation of the Photocatalytic Activity of a Cordierite-Honeycomb-Supported TiO_2_ Film with a Liquid–Solid Photoreactor

**DOI:** 10.3390/molecules24244499

**Published:** 2019-12-09

**Authors:** Francesco Pellegrino, Nicola De Bellis, Fabrizio Ferraris, Marco Prozzi, Marco Zangirolami, Jasmine R. Petriglieri, Ilaria Schiavi, Alessandra Bianco-Prevot, Valter Maurino

**Affiliations:** 1 Department of Chemistry and NIS Inter-Departmental Centre, University of Torino, 10125 Torino, Italy; francesco.pellegrino@unito.it (F.P.); nicola.debellis@unito.it (N.D.B.); fabrizio.ferraris@edu.unito.it (F.F.); ilaria.schiavi@unito.it (I.S.); valter.maurino@unito.it (V.M.); 2 Fonderia Mestieri S.r.l, 10093 Collegno TO, Italy; marco.zangirolami@fonderiamestieri.com; 3 Department of Chemistry and G. Scansetti Center, University of Torino, 10125 Torino, Italy; jasminerita.petriglieri@unito.it

**Keywords:** titanium dioxide, photoreactor, cordierite, sulfates, photocatalysis, wastewater

## Abstract

Anatase nanoparticles in suspension have demonstrated high photoactivity that can be exploited for pollutant removal in water phases. The main drawback of this system is the difficulty of recovering (and eventually reusing) the nanoparticles after their use, and the possible interference of inorganic salts (e.g., sulfates) that can reduce the performance of the photocatalyst. The present work describes the development of a cordierite-honeycomb-supported TiO_2_ film to eliminate the problems of catalyst recovery. The catalyst was then tested against phenol in the presence of increasing concentrations of sulfates in a specially developed recirculating modular photoreactor, able to accommodate the supported catalyst and scalable for application at industrial level. The effect of SO_4_^2−^ was evaluated at different concentrations, showing a slight deactivation only at very high sulfate concentration (≥3 g L^−1^). Lastly, in the framework of the EU project Project Ô, the catalyst was tested in the treatment of real wastewater from a textile company containing a relevant concentration of sulfates, highlighting the stability of the photocatalyst.

## 1. Introduction

Titanium dioxide (TiO_2_) is a wide-band-gap semiconductor largely employed in several multisectorial applications due to its interesting functional properties coupled with a high chemical and physical stability, together with low production cost and negligible toxicity [1,2,3]. Under suitable irradiation, TiO_2_ behaves as a photocatalyst, generating electron-hole couples able to react with different substrates present at its surface [4,5,6,7]. In particular, in aqueous phase, holes can oxidize water molecules generating OH radicals or directly oxidize adsorbed substrates [8]. This feature has been extensively studied in the last decades for environmental remediation, i.e., the degradation/mineralization of organic pollutants present in gas or liquid phases [9]. In water treatment, TiO_2_ is usually suspended in the waste stream [10,11]; in this way, a good contact between catalyst surface and the fluid phase is achieved. On the other hand, the suspension implies the necessity of a separation stage after the treatment in order to recover the catalyst and eventually re-use it. For this reason, the possibility of immobilizing the photocatalyst on a support has been widely explored in the literature [9,12,13,14,15]. TiO_2_ has been deposited on different materials that can also actively participate to the photocatalytic process [16,17]. The supports ensure easy reuse of the material, allowing the scale-up of the photocatalytic treatment to industrial levels [18]. However, reactors based on immobilized catalysts have a lower area/volume ratio, which can lead to mass-transfer limitations [19].

In this work, the colloidal synthesis of TiO_2_ nanoparticles was developed in order to obtain the active material. A cordierite honeycomb monolith was employed as solid, inert substrate for TiO_2_ deposition to obtain a thin-film-coated catalyst able to remove pollutants from an aqueous phase. The honeycomb structure can provide a relatively large area covered with a TiO_2_ film, which can be irradiated with diffuse light. Different examples of immobilized TiO_2_ reactors can be found in literature [20,21], but very few have utilized a monolithic supported photocatalyst for water treatment. In the present work, the photocatalytic activity of the cordierite-honeycomb-supported TiO_2_ film was tested in a liquid–solid reactor developed in the framework of the H2020 Project Ô as a bench scale reactor to be implemented in a modular water treatment system used for the purification of industrial wastewaters from textile industry [22]. This system for water treatment, if correctly implemented, could allow the recycling of industrial water in selected facilities (e.g., textile industries), allowing circular economy goals for sustainable water management to be reached [23]. Phenol was used as the target molecule since its photodegradation mediated by TiO_2_ has been extensively studied. Special attention was given to the effect of sulfate ions on the process, since high amounts of sulfate are usually present in wastewater from textile industry. It has been reported that sulfates can interfere with the degradation of organic matter in TiO_2_ photocatalysis, mostly in acidic environments and at low concentrations [24]. This effect has been explained by the immediate adsorption of inorganic salts on the titania surface to form a layer that avoids reaction with the organic substrate [25]. Some other works have suggested that sulfate, after an initial decrease due to adsorption, could have a slightly enhancing effect due to the generation of sulfate radicals under illumination [26]. 

## 2. Results and Discussion

### 2.1. Colloid and Film Characterization

**Figure 1:**
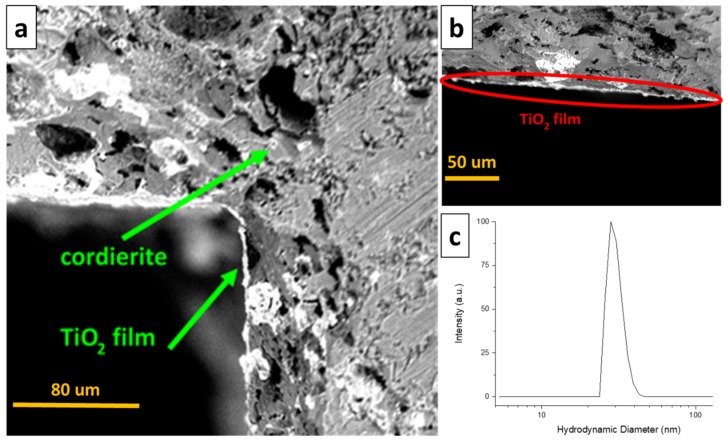
Scanning electron microscope (SEM) micrographs of the TiO_2_ film on the cordierite honeycomb (**a**,**b**) and hydrodynamic diameter distribution (by dynamic light scattering, DLS) of the colloidal TiO_2_ nanoparticles obtained from the colloidal synthesis (**c**).

The colloidal synthesis of TiO_2_ allowed a stable colloid constituted of amorphous nanoparticles with a narrow size distribution around 25 nm to be obtained (Figure 1c). The uniformity of the nanoparticles and the colloidal stability allow a TiO_2_ film on the cordierite honeycomb of around 3 um thickness to be coated, as highlighted in Figure 1a,b.

**Figure 2:**
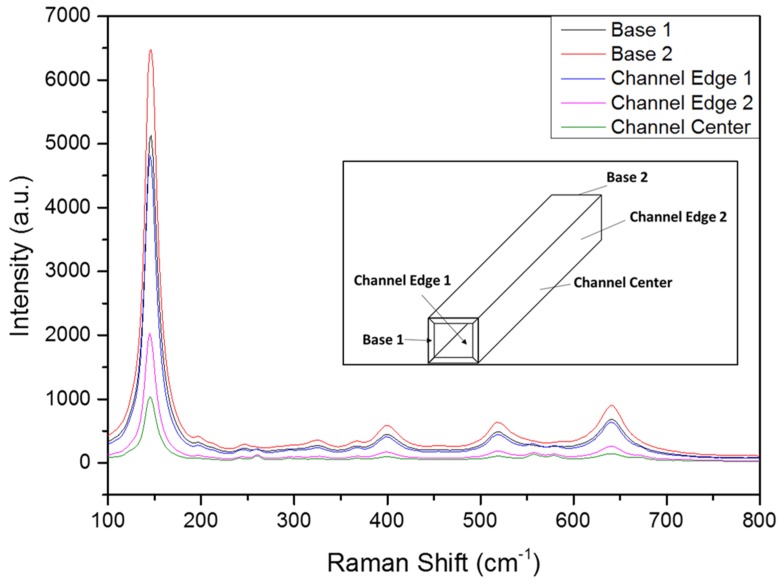
Micro-Raman spectra obtained in different zone of the cordierite monolith. Base 1 and 2 refers to the Raman spectra obtained on the edge of the channels obtained at the two bases of the monolith. The spectra labeled Channel are obtained inside the channels. Inset: reference of the zones in which the Raman analyses were carried out.

In order to determine the TiO_2_ phase and the uniformity of the coating, Raman analysis was carried out on different zones of the cordierite honeycomb channel (Figure 2).

Raman spectra revealed that the TiO_2_ phase was anatase [27]. No peaks of rutile or brookite were detected. Moreover, from the Raman intensity of the anatase signals and the presence of the cordierite Raman lines, an inhomogeneity of the anatase layer was apparent. The intensity of the 150 cm^−1^ E_g_ Raman line of the anatase layer at the centre of the channel was roughly one sixth of the intensity at the base edges and the cordierite doublet at 555/576 cm^−1^ appeared sharply, hardly discernible in the Raman spectra of the edges (see inset of Figure 2).

### 2.2. Photocatalytic Tests

The photocatalytic tests were carried out using phenol as a probe molecule. Phenol is one of the most employed substrates for the evaluation of the photoactivity of TiO_2_ in aqueous environments [28,29,30]. Before turning on the light, 20 min of recirculation in dark of the phenol solution inside the reactor was carried out, highlighting that a negligible absorption effect occurred. No leaching of TiO_2_ was observed even after several photocatalytic tests. The ultimate purpose of the entire photocatalytic system is the mineralization of the organic content of a real wastewater coming from the drains of a textile industry, in which sulfates are the predominant anion. Therefore, the experiments were directed to evaluate the effect of sulfates on the phenol degradation rate performing photocatalytic tests at increasing amounts of SO_4_^2−^ (from 0 to 7 g L^−1^). It has been reported that sulfates could have a detrimental effect on the photocatalytic activity of TiO_2_ nanoparticles due to the strong adsorption of the sulfates ions on titania surface, limiting the interaction with the organic matter [24]. 

**Figure 3:**
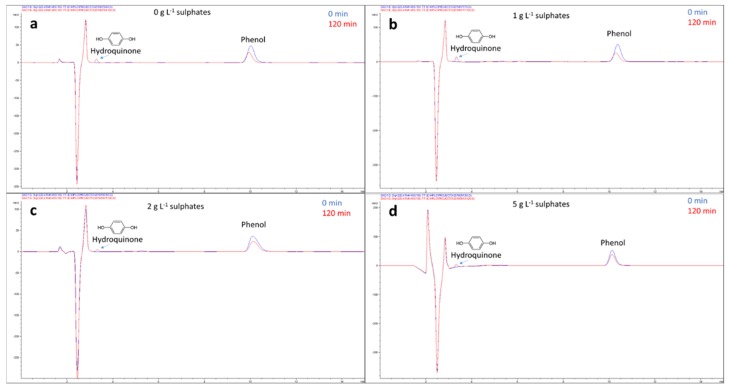
Chromatograms at 0 and 120 min of the phenol degradation at different concentrations of sulfate: (**a**) 0 g L^−1^, (**b**) 1 g L^−1^, (**c**) 2 g L^−1^, and (**d**) 5 g L^−1^.

In Figure 3, as examples, the chromatograms (before and after 120 min of irradiation) of four selected tests carried out at different concentrations of sulfates are reported. Hydroquinone was the only intermediate found in all the tests. 

**Figure 4:**
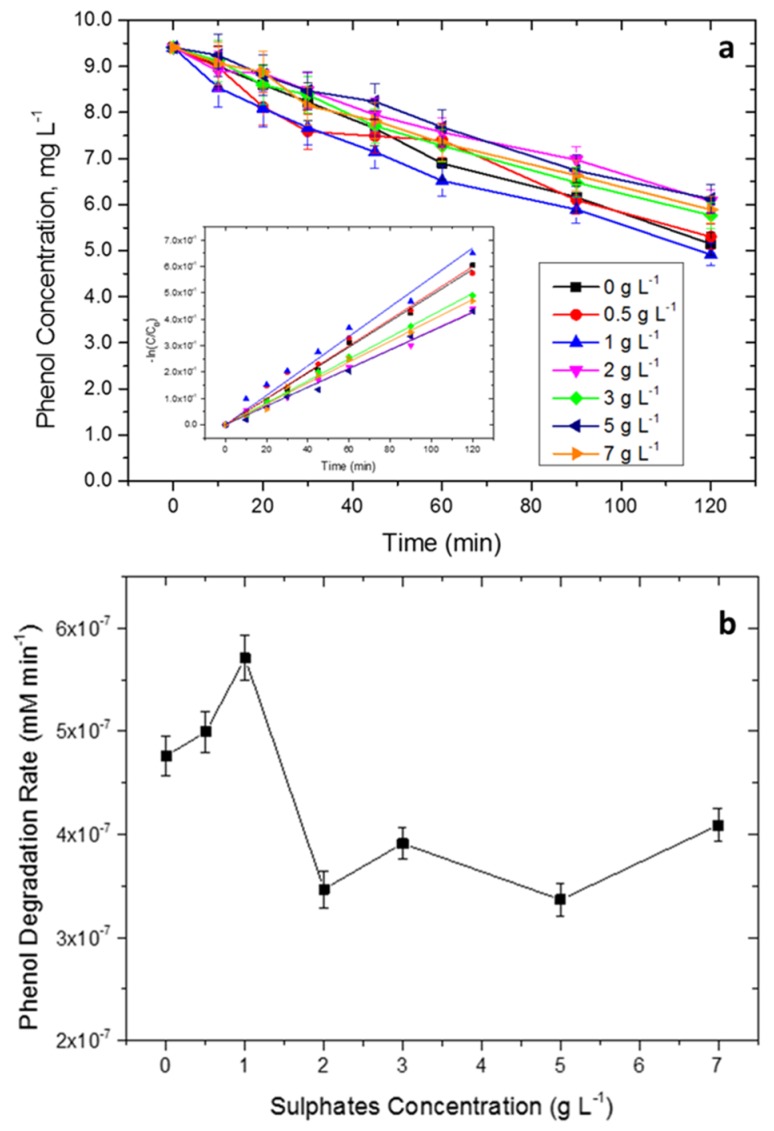
Degradation curves of phenol abatement on the cordierite-supported TiO_2_ film at different sulfate concentrations (**a**) (inset: fit for first order kinetics) and phenol degradation rate at different sulfate concentrations (**b**).

Figure 4 shows the degradation curves and the photocatalytic rates of phenol abatement on the cordierite-supported TiO_2_ film at different sulfate concentrations. A first-order kinetic was used for all the phenol abatement trends (inset in Figure 4a) to obtain the initial phenol degradation rate reported in Figure 4b. 

From Figure 4, it is possible to see that the effect of sulfates on the phenol was not univocal. Indeed, although a maximum of activity was reached at 1 g L^−1^ of SO_4_^2−^, a further increase of the sulfate concentration decreased the activity of the catalyst. This behavior can be ascribed to the competition of two different mechanisms: at low concentrations (1 g L^−1^), sulfate reaction with TiO_2_ can lead to the production of the radical SO_4_^2−^ (through hole transfer), which can participate in the phenol degradation, increasing the abatement rate [31,32,33,34]. On the other hand, increasing the [SO_4_^2−^], the previously generated sulfate radical anion can be in turn reduced by photogenerated electrons due to the higher adsorption on the semiconductor surface; this adsorption can also reduce the interaction with the substrate [33,34]. The decrease of the photocatalytic activity was decidedly less evident compared to the tests carried out on TiO_2_ suspensions in literature [24]. In this latter case, a second effect could influence the degradation rate: the suspended nanoparticles tend to agglomerate in the presence of inorganic salts, reducing the available surface area [35]. Moreover, the agglomeration could change the optical properties of the nanoparticles, reducing their photocatalytic activity [28]. These detrimental effect were absent in the case of supported catalyst.

**Figure 5:**
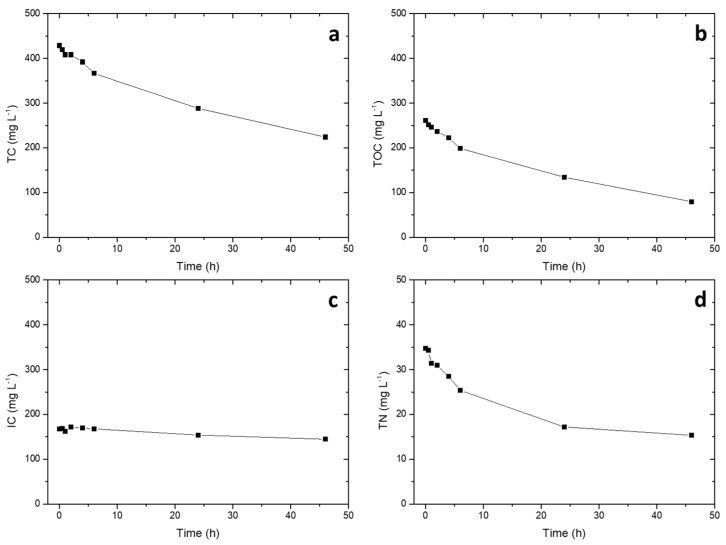
Temporal trends of total carbon (**a**), total organic carbon (**b**), inorganic carbon (**c**), and total nitrogen (**d**) during the photocatalytic test on the real wastewater.

Given the good effectiveness on phenol degradation in the presence of high concentration of sulfates, the photocatalytic system was finally tested against the real wastewater (0.15 g L^−1^, pH 6.5). At this concentration of SO_4_^2−^, based on data reported in Figure 4, a negligible effect of sulfates was hypothesized; the developed catalyst could therefore be optimal for the treatment. The photocatalytic performance was evaluated monitoring several parameters, in particular the trends of the total organic carbon (TOC) and the total nitrogen (TN) during irradiation (Figure 5b). A decrease of the TOC of 70% in 46 h of irradiation was observed, while the decrease of the total nitrogen (TN) settled at 55%. The trend of the IC (inorganic carbon) was nearly constant during all the photodegradation test; at pH 6.5, the main inorganic species were bicarbonates (HCO_3_^−^), which can slightly decrease the electron–hole recombination, increasing the degradation rate of the catalyst [36,37].

The results highlighted in Figure 5 confirm the suitability of the developed system (catalyst + photoreactor) for the treatment of industrial wastewater.

Table 1 reports the main composition parameters of the wastewater before and after the photocatalytic treatment.

## 3. Materials and Methods

### 3.1. TiO_2_ Nanoparticle Synthesis

Colloidal TiO_2_ solutions were prepared from titanium(IV) isopropoxide (TIPO, 97% by Sigma Aldrich, Darmstadt, Germany) as a precursor. A 0.1 M HCl (37%, by Sigma Aldrich) aqueous solution in a triple-neck, round-bottomed flask was placed in a fridge below 0 °C for 45 min. The required amount of TIPO was added dropwise (i.e., 7–8 mL min^−1^) to the acidic solution under vigorous stirring and N_2_ atmosphere/flow. The TIPO exothermic hydrolysis induced a turbid solution that was heated up until 20 °C at the end of this addition. The flask was then heated up to 80 °C and kept at this temperature for about 18 h under vigorous stirring in order to reach complete peptization of the precursor. The volume of the suspension was maintained constant during the synthesis by adding aliquots of ultrapure water (Milli-Q). Finally, at the end of the peptization process, the white suspension was homogenized by 2 h sonication in a glass bottle. The synthetized amorphous TiO_2_ nanoparticles had a final nominal concentration of 100 g L^−1^ and were stored at room temperature.

### 3.2. Photocatalyst Immobilization on the Cordierite Honeycomb Support

**Figure 6:**
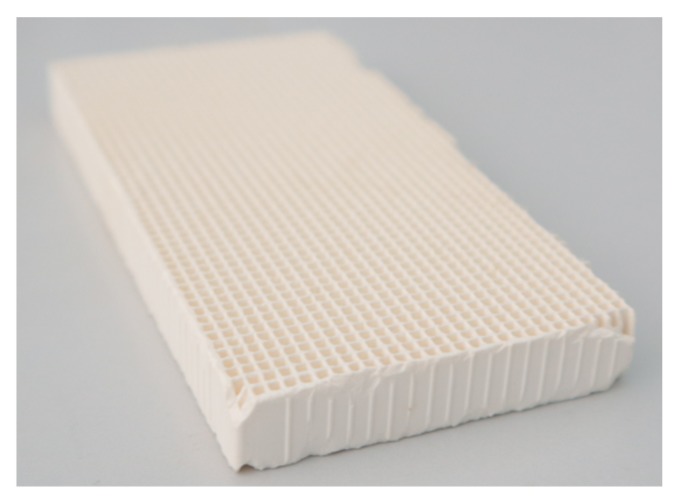
The cordierite honeycomb monolith utilized in the photoreactor. Dimensions: 10 × 5 × 1 cm; 1456 channels: 0.17 × 0.17 cm.

TiO_2_ colloid was immobilized using a honeycomb cordierite monolith as a support (Figure 6). The monolith was first washed with Milli-Q water and calcined at 600 °C for 1 h in air in order to remove any possible organic and inorganic contaminants. The catalyst deposition was then performed by dip-coating the ceramic substrate into the suspension of TiO_2_ for 15 min. The support was immediately dried at 120 °C for 1 h, and finally treated at high temperatures (600 °C). This ensured the crystallization of the amorphous TiO_2_ colloids into a nanocrystalline active anatase form suitable for the photo-oxidation processes. The final amount of deposited TiO_2_ was nearly 1.5 g. The procedure as eventually repeated multiple times to increase the TiO_2_ washcoat thickness. The supported photocatalysts thus prepared were labeled and ready to be tested in the degradation.

### 3.3. Dynamic Light Scattering (DLS) Analysis of the Colloidal Nanoparticles

In order to estimate the size of the synthetized TiO_2_ nanoparticles, hydrodynamic diameters were evaluated. The DLS analysis was carried out with a Cilas Nano DS particle size analyzer. Each suspension was diluted to 20 mg L^−1^ in HClO_4_ 10^−3^ M and sonicated for 1 h before analysis. 

### 3.4. Scanning Electron Microscopy (SEM) Analysis

SEM analysis was necessary to evaluate the formation of the washcoat on the cordierite support after the deposition of the catalyst. After preparation, the photocatalytic monolith was cut perpendicularly to the directions of the channels. This microscopic characterization was performed using a Phenom Pro Desktop SEM by Thermo Fisher Scientific (Waltham, MA, USA).

### 3.5. Raman Spectroscopy Analysis

Micro-Raman analysis was carried out in nearly backscattered geometry with a Horiba JobinYvon HR800 spectrometer equipped with an Olympus microscope with an objective up to 100×, two gratings 1800 and 600 grooves/mm, a cooled CCD detector (−70 °C), and two polarized lasers: a red HeNe laser (wavelength 632.8 nm, power 20 mW) and an Nd solid-state green laser (wavelength 532 nm, power 250 mW). The system was completed with Edge filters for laser lines 633 nm and 532 nm and a set of interference filters. Spectral resolution with the grid in 1800: 2 cm^−1^ for green lasers and 1.5 cm^−1^ for red laser. The system was calibrated using the 520.6 cm^−1^ Raman peak of silicon before each experimental session. The spectra were collected using the 100× objective with repeated acquisition: three acquisitions for 20 s.

### 3.6. Photodegradation Experiments

**Figure 7:**
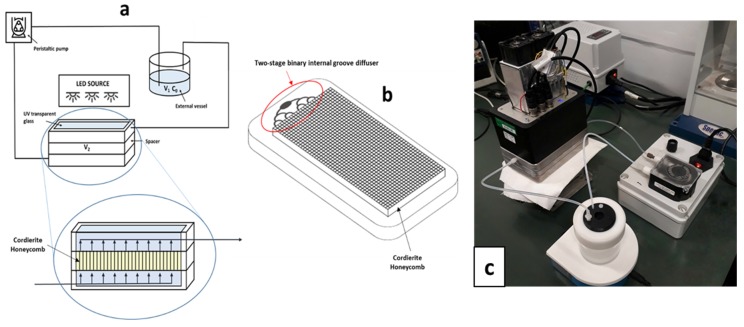
Scheme of the developed photoreactor with a focus on the reaction chamber (**a**) and its two-stage binary internal groove diffuser used for the flow homogenization (**b**). Picture of the real photoreactor (**c**).

A flow-through bench scale reactor was developed (Figure 7a). It consisted of an aluminium case protected with a TEFLON^®^ AF coating to prevent the release of Al ions which can interfere with the photocatalytic process. In the case, a two stage binary internal grove diffuser (both at the inlet and at the outlet) was milled to obtain a homogeneous flow. It was equipped with two 3.3 mm thick, UV transparent glasses and a spacer where an approximately 10 × 5 × 1 cm cordierite monolith could be placed; the inner reactor volume was approximately 100 mL. The reactor was clamped with a set of screws. Externally, a 50 mL Teflon vessel was connected through inert tubes that could be inserted in the vessel cap, where a cavity allowed the sampling and, eventually, the continuous monitoring of pH and dissolved O_2_. When the photocatalytic material is immobilized on a support through which contaminated water is passed, there is a short contact time between the photocatalyst and pollutant molecules, resulting in poor performance. This is why it a multiple stage or recirculating system is usually used to achieve good degradation efficiencies [38]. Hence, a maximum of 150 mL of aqueous solution could be treated in continuous-recycling mode thanks to a peristaltic pump with a flux rate up to 100 mL/min.

The irradiation apparatus consisted of two UV 360 nm LEDs (model LZ1-00UV00-0100) and a system for temperature control. The LEDs were mounted on a printed circuit and driven with a constant current power supply with a feedback control on irradiance. The radiation was filtered with two 3.3 mm thick frosted borosilicate glasses in order to obtain a uniform diffuse radiation. The illuminator was placed on the reactor’s transparent windows to irradiate the photocatalyst while the solution flowed through the reaction chamber. Thus, an active volume of approximately 100 mL was continuously illuminated. To distribute the solution flow uniformly through the reaction chamber, a two-stage binary diffusion internal groove was carved in both parts of the case to let the flow in and out (Figure 7b). 

Before the degradation, ultrapure water without substrate was recirculated through the reactor under UV illumination for 40 min to remove any possible presence of organic substances that could compete with the substrate. The light was then turned off and the phenol (99% by Sigma Aldrich) was added, reaching a final concentration of 0.1 mM. Once the system had reached homogeneity, the UV lamp (irradiation 45 W m^−2^ centred 365 nm) was again turned on and the degradation started. The irradiation time for the phenol abatement was fixed to 120 min. Each experiment was repeated three times in order to estimate the repeatability of the experiment. The pH of the solution was 6.5 and was unaffected by phenol addition. The solution was sampled from the external vessel at different times and analyzed. In the tests carried out at different sulfate concentrations, Na_2_SO_4_ (99% by Sigma Aldrich) was used. For the test carried out on the real wastewater, a 20 min equilibration was done. The irradiation time for the wastewater treatment was fixed to 48 h. All experiments were carried out by using the same photocatalyst, which showed a negligible decrease in photoactivity after 500 h of irradiation.

### 3.7. High-Performance Liquid Chromatography Analysis

HPLC determination of phenol was carried out with an Agilent Technologies (Santa Clara, California, USA) HPLC chromatograph 1200 Series equipped with a diode array detector, binary gradient high-pressure pump, and an automatic sampler. Isocratic elution was carried out with a mixture of 20/80 acetonitrile/formic acid aqueous solution (0.1% w/v), a flow rate 0.5 mL min^−1^, and injection volume 20 μL. The column used was a LiChrospher^®^ 100 RP-18 (5 µm) LiChroCART^®^ 125-4.

### 3.8. Total Organic Carbon and Total Nitrogen Analysis

Total organic carbon (TOC) was obtained from the difference between total carbon (TC) and inorganic carbon (IC) for real (not synthetic) water samples using a Shimadzu TOC-5000 analyzer (catalytic oxidation with Pt at 680 °C, NDIR method), equipped with an ASI-V autosampler and fed with zero-grade air. Total nitrogen analysis were possible thanks to the presence of a TNM-1 unit (720 °C catalytic oxidation, chemiluminescence method).

### 3.9. Wastewater Samples

The wastewater was sampled from the mixed effluent of the dyeing and washing baths of a textile plant after primary clarification. The clarification was done by adding polyelectrolytes and ferric sulfate. The company was Galeb d.d., located in Omis (Croatia), and it is a demonstration site of the E.U. project “ProjectO”. The wastewater was filtered onsite after sampling on a 0.45 micron cellulose acetate membrane (HA Millipore) and kept frozen until use. Chemical analysis data are reported in Table 1. The main wastewater organic contaminants present were non-ionic and anionic surfactants.

## 4. Conclusions

In conclusion, this work demonstrated the possibility of using cordierite honeycomb as a support for TiO_2_ film, eliminating the problems of suspended catalyst recovery. The peculiar structure of the support minimized the loss of surface area due to the catalyst immobilization. The synthetized photocatalyst, tested at different concentration of sulfates, showed a maximum of photoactivity at [SO_4_^2−^] lower than 3 g L^−1^, likely due to generation of SO_4_^2^^−^ which can increase the oxidation of organic substrate. At higher concentration of sulfates, only a slight decrease of the activity occurred due to the irrelevance of catalyst agglomeration that, together with adsorption, is the main reason of the decreased activity for suspended TiO_2_ systems. The suitability of the system was confirmed against real wastewater characterized by a discrete amount of sulfates; the total organic carbon present was reduced of 70% in 46 h of irradiation. This results encourage the implementation of this system at industrial scales in the treatment of wastewater. Indeed, two or more cordierite-supported TiO_2_ film modules could be used in series in a scaled-up photoreactor that could lead to a fast, simple, and cheap abatement of organic pollutants.

## Figures and Tables

**Table 1:** Selected analytical parameters of the wastewater before and after photocatalytic treatment.

Time	Hardness	pH	Chlorides, mg L^−1^	Sulfates, mg L^−1^	Conductivity	Suspended Matter
0 h	7 °F	9.05	2.3	155	2.01 mS cm^−1^	Sample filtered, <1 mg/L
48 h	7 °F	7.98	2.5	156	1.82 mS cm^−1^	Sample filtered, <1 mg/L
